# SARS-CoV-2 Infection and Pregnancy: Maternal and Neonatal Outcomes and Placental Pathology Correlations

**DOI:** 10.3390/v14092043

**Published:** 2022-09-14

**Authors:** Michał Pomorski, Martyna Trzeszcz, Agnieszka Matera-Witkiewicz, Magdalena Krupińska, Tomasz Fuchs, Mariusz Zimmer, Aleksandra Zimmer-Stelmach, Anna Rosner-Tenerowicz, Joanna Budny-Wińska, Anna Tarczyńska-Podraza, Klaudia Radziejewska, Barbara Królak-Olejnik, Anna Szczygieł, Hanna Augustyniak-Bartosik, Magdalena Kuriata-Kordek, Karolina Skalec, Izabela Smoła, Ewa Morgiel, Jakub Gawryś, Adrian Doroszko, Piotr Rola, Małgorzata Trocha, Krzysztof Kujawa, Barbara Adamik, Krzysztof Kaliszewski, Katarzyna Kiliś-Pstrusińska, Marcin Protasiewicz, Janusz Sokołowski, Ewa A. Jankowska, Katarzyna Madziarska

**Affiliations:** 1 Clinical Department of Gynecology and Obstetrics, Wroclaw Medical University, Borowska Street 213, 50-556 Wroclaw, Poland; 2 Division of Pathology and Clinical Cytology, University Hospital in Wroclaw, Borowska Street 213, 50-556 Wroclaw, Poland; 3 Screening of Biological Activity Assays and Collection of Biological Material Laboratory, Wroclaw Medical University Biobank, Faculty of Pharmacy, Wroclaw Medical University, Borowska Street 211A, 50-556 Wroclaw, Poland; 4 Department of Neonatology, Wroclaw Medical University, Borowska Street 213, 50-556 Wroclaw, Poland; 5 Clinical Department of Nephrology and Transplantation Medicine, Wroclaw Medical University, Borowska Street 213, 50-556 Wroclaw, Poland; 6 University Hospital in Wroclaw, Borowska Street 213, 50-556 Wroclaw, Poland; 7 Clinical Department of Gastroenterology and Hepatology, Wroclaw Medical University, Borowska Street 213, 50-556 Wroclaw, Poland; 8 Clinical Department of Rheumatology and Internal Medicine, Wroclaw Medical University, Borowska Street 213, 50-556 Wroclaw, Poland; 9 Clinical Department of Internal Medicine, Hypertension and Clinical Oncology, Wroclaw Medical University, Borowska 213, 50-556 Wroclaw, Poland; 10 Department of Cardiology, Provincial Specialized Hospital, Iwaszkiewicza Street 5, 59-220 Legnica, Poland; 11 Department of Pharmacology, Wroclaw Medical University, Mikulicz-Radecki Street 2, 50-345 Wroclaw, Poland; 12 Statistical Analysis Centre, Wroclaw Medical University, K. Marcinkowski Street 2-6, 50-368 Wroclaw, Poland; 13 Clinical Department of Anaesthesiology and Intensive Therapy, Wroclaw Medical University, Borowska Street 213, 50-556 Wroclaw, Poland; 14 Clinical Department of General, Minimally Invasive and Endocrine Surgery, Wroclaw Medical University, Borowska Street 213, 50-556 Wroclaw, Poland; 15 Clinical Department of Paediatric Nephrology, Wroclaw Medical University, Borowska Street 213, 50-556 Wroclaw, Poland; 16 Clinical Department and Clinic of Cardiology, Wroclaw Medical University, Borowska Street 213, 50-556 Wroclaw, Poland; 17 Department of Emergency Medicine, Wroclaw Medical University, Borowska Street 213, 50-556 Wroclaw, Poland; 18 Institute of Heart Diseases, University Hospital, Borowska Street 213, 50-556 Wroclaw, Poland; 19 Department of Translational Cardiology and Clinical Registries, Wroclaw Medical University, Pasteura Street 1, 50-367 Wroclaw, Poland

**Keywords:** SARS-CoV-2, COVID-19, vertical transmission, pregnancy, maternal and neonatal outcome, placental pathology

## Abstract

There is accumulating evidence on the perinatal aspects of COVID-19, but available data are still insufficient. The reports on perinatal aspects of COVID-19 have been published on a small group of patients. Vertical transmission has been noted. The SARS-CoV-2 genome can be detected in umbilical cord blood and at-term placenta, and the infants demonstrate elevated SARS-CoV-2-specific IgG and IgM antibody levels. In this work, the analysis of clinical characteristics of RT-PCR SARS-CoV-2-positive pregnant women and their infants, along with the placental pathology correlation results, including villous trophoblast immunoexpression status for SARS-CoV-2 antibody, is presented. RT-PCR SARS-CoV-2 amniotic fluid testing was performed. Neonatal surveillance of infection status comprised RT-PCR testing of a nasopharyngeal swab and the measuring of levels of anti-SARS-CoV-2 in blood serum. In the initial study group were 161 pregnant women with positive test results. From that group, women who delivered during the hospital stay were selected for further analysis. Clinical data, laboratory results, placental histomorphology results, and neonatal outcomes were compared in women with immunohistochemistry (IHC)-con SARS-CoV-2-positive and IHC SARS-CoV-2-negative placentas (26 cases). A positive placental immunoprofile was noted in 8% of cases (*n* = 2), whereas 92% of cases were negative (*n* = 24). Women with placental infection proven by IHC had significantly different pathological findings from those without. One infected neonate was noted (*n* = 1; 4%). Infection was confirmed in perinatal autopsy, as there was the intrauterine fetal demise. The potential course of the infection with the risk of vertical transmission and implications for fetal–neonatal condition is critical for proper clinical management, which will involve comprehensive, multidisciplinary perinatal care for SARS-CoV-2-positive patients.

## 1. Introduction

In December 2019, a series of pneumonia cases were observed in Wuhan City in China; then, a severe acute respiratory syndrome coronavirus 2 (SARS-CoV-2) was identified as a new human pathogen [1]. The first patient infected in Poland was diagnosed on 4 March 2020. The first step was to protect the elderly and patients with comorbidities. It was unknown how the SARS-CoV-2 infection could affect the clinical condition of pregnant women and the condition of newborns. There is still no clear proof for possible maternal–fetal transmission and infant infection during delivery [2,3]. Current research confirms that, although rare, SARS-CoV-2 transmission in utero is possible [4,5,6]. According to the definition formulated by an international multidisciplinary panel of experts appointed by WHO, mother-to-child transmission can be differentiated to in utero transmission, intrapartum transmission, and postnatal transmission. In utero transmission occurs when the pathogen crosses the maternal–placental interface. Intrapartum transmission occurs during labor and childbirth. Postnatal transmission occurs through breastfeeding via the human milk or through contact with infected caregiver through the respiratory system [6,7]. There are several studies which focused on the analyses of the viral genome from nasopharyngeal swabs from mothers and infants; and umbilical cord plasma, placenta, umbilical cord biopsies, amniotic fluid, and milk. The SARS-CoV-2 genome can be detected in umbilical cord blood, and at-term placentas and the infants also demonstrated elevated SARS-CoV-2-specific IgG and IgM antibody levels [4]. However, the transfer of IgM across the placenta is rare. In turn, IgG antibodies can be detected in the cord blood of 87–90% of neonates born to a previously infected mother with positive serology at the time of delivery [8]. Noteworthily, in mothers vaccinated during pregnancy, the increased levels of IgG antibodies have been detected in newborns up to 3 months after birth, confirming that the antibodies can cross the placental barrier and protect neonates from infection [9].

Placental pathology examination performed by a qualified perinatal pathologist plays a fundamental role in identifying causes of adverse perinatal outcomes and in improving understanding of their pathophysiology [10]. Cytomegalovirus and herpes simplex virus are the most commonly morphologically manifested viral placental infections. Clinical features include chronic lymphoplasmacytic villitis, characteristic inclusions, and hemosiderin villous depositions with or without associated intervillositis [11]. The inflammatory histopathologic features identified in SARS-CoV-2-positive mothers with evidence of placenta infection seem to be similar to those of other RNA virus infections of pregnancy; however, findings consistent with acute chorioamnionitis have also been reported [12]. Placental immunohistochemistry is one of the reported unequivocal biomarkers of identification of SARS-CoV-2’s presence in the placental tissue and transplacental infection [6]. The highest specificity and sensitivity are achieved using antibodies against the viral nucleocapsid [13]. Identification of viral particles in tissue samples by electron microscopy is questionable due to a large number of potential errors in evaluation and misinterpretation. Refined criteria for optimized sampling and ultrastructural analysis have been proposed [13].

Current scientific evidence supports no pathognomic histomorphology in second and third trimester placentas from SARS-CoV-2-infected mothers [14]. Reported placental lesions in maternal SARS-CoV-2 infection have recognizable histomorphologic features. Maternal vascular malperfusion (MVM), primarily caused by abnormal uterine perfusion, and subsequent hypoxic-ischemic placental injury, has the highest incidence among placental pathology findings according to the recent large reviews and meta-analyses [12,15]. Placental inflammatory changes occupy at most second place in incidence. Fetal vascular malperfusion (FVM), primarily caused by impaired placental blood supply, has been also reported in maternal cases of COVID-19 [12,16,17]. MVM and FVM may reflect non-specific placental findings [18], as these could be associated with gestational and chronic hypertension, systemic lupus erythematosus, inherited thrombophilias, and coagulation disorders—numerous maternal conditions. MVM may be present in mothers without any underlying disease [19]. Further placental investigations need to be conducted along with a careful interpretation of results by the perinatal multidisciplinary teams.

Few data exist regarding possible implications of maternal SARS-CoV-2 infection for the fetus or neonate, such as effects on placenta function and the frequency of placental infection, and available data are based on a small sample size [17,20,21,22]. In this post hoc study on perinatal aspects of the SARS-CoV-2 pandemic, we analyzed clinical characteristics of RT-PCR SARS-CoV-2-positive pregnant women and their infants along with the placental pathology correlation results, including villous trophoblast immunoexpression status for SARS-CoV-2 antibody. 

## 2. Materials and Methods

### 2.1. Study Population and Procedures

**Figure 1:**
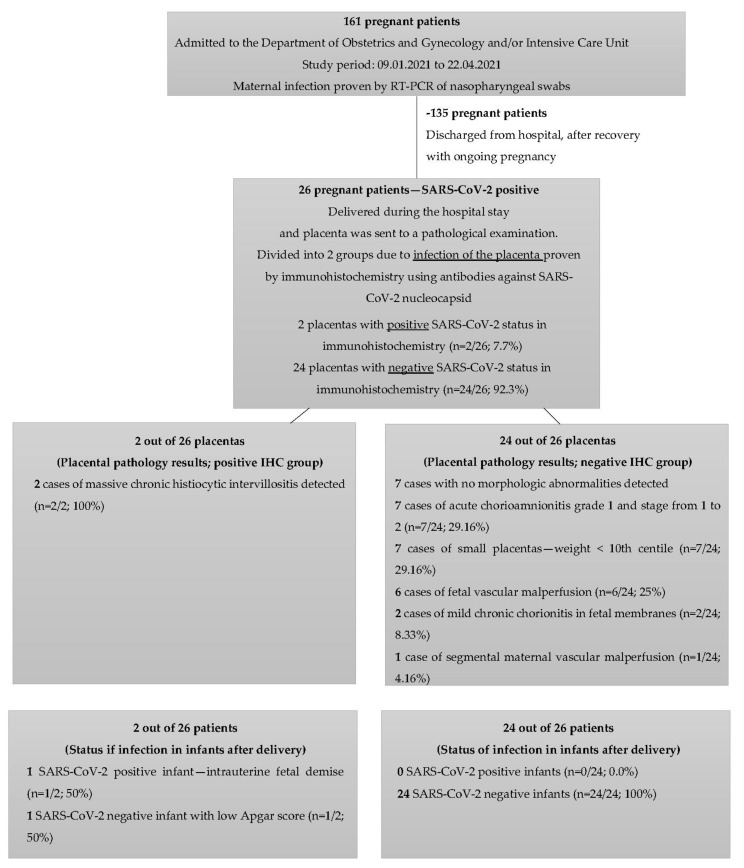
Flowchart presenting the patients enrolled in the study.

The initial study group included 161 pregnant women with positive test results for SARS-CoV-2 using reverse transcription polymerase chain reaction (RT-PCR) and nasopharyngeal swabs, who were admitted to the Department of Obstetrics and Gynecology and/or Intensive Care Unit of the University Hospital in Wroclaw, Poland, in 2021. One-hundred and thirty-five patients were discharged from the hospital with ongoing pregnancy. Twenty-six patients who delivered during the hospital stay were selected for further groups comparisons. Data were collected from patients’ medical electronic records, allowing the assessment of maternal and neonatal outcomes with postnatal infection status. Below, the flowchart with patients enrolled in the study is presented (Figure 1).

The RT-PCR for SARS-CoV-2 was performed on all pregnant women upon admission to the hospital. The procedure of collecting amniotic fluid at the time of cesarean section consisted of puncturing the amniotic sack and aspirating 20 mL of amniotic fluid directly after the uterotomy and before fetal extraction. During the first stage of vaginal delivery, 20 mL of amniotic fluid was collected in cases of preterm rupture of membranes from the vagina during the speculum examination and in cases of intact membranes at the time of amniotomy. Samples contaminated by blood were excluded. Maternal venous blood for anti-SARS-CoV-2 IgG assessment was collected at the time of delivery. Neonatal surveillance of SARS-CoV-2 infection comprised RT-PCR testing of a nasopharyngeal swab and assessment of anti-SARS-CoV-2 IgG in blood serum. The nasopharyngeal swabs of the infants were collected within the first 24 h after delivery. Fetal blood for anti-SARS-CoV-2 IgG assessment was collected at the time of delivery, by puncture of the umbilical vein immediately after clamping and cutting the umbilical cord. Laboratory tests performed in the SARS-CoV-2-positive group of pregnant women included in this study were as follows: white blood cell count, hemoglobin, C-reactive protein (CRP), platelets (PLT), aspartate aminotransferase (AST), alanine aminotransferase (ALT), and activated partial thromboplastin time (aPTT). All patients received low-molecular-weight heparin in doses adjusted to body weight during the hospital stay. In cases of planned deliveries, the administration was stopped 12 h before delivery and resumed 12 h after.

Demographic variables collected were maternal age, gestational age, SpO2 levels during admission and delivery, and mode and indications for delivery. Exclusion criteria were as follows: ongoing pregnancy at discharge from the hospital, absent histopathological examination of the placenta, and missing clinical data.

The final study group consisted of 26 patients who delivered a child at the university hospital in Wroclaw during the hospital stay and were not excluded from the study. Patients were divided into 2 groups: with positive IHC SARS-CoV-2 of placenta and negative IHC SARS-CoV-2 of placenta.

### 2.2. Placental Pathology and SARS-CoV-2 Immunoexpression Studies

**Figure 2:**
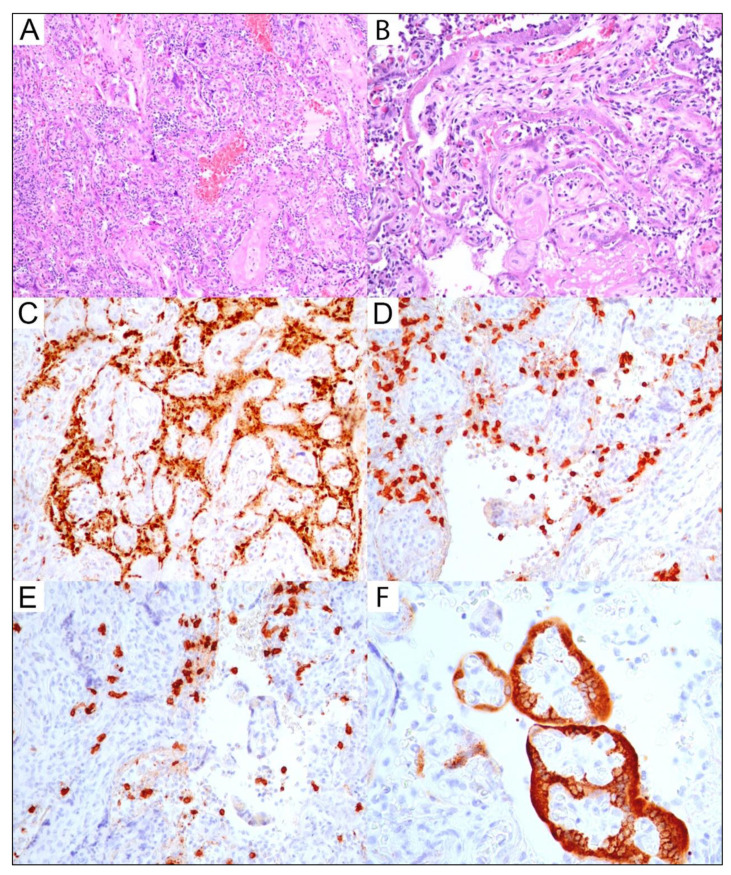
Severe pathological findings identified in a placenta with a positive SARS-CoV-2 immunoprofile from a SARS-CoV-2-positive mother. Neonate had a result positive result for a nasopharyngeal swab. Massive chronic intervillositis: (**A**): Intervillous space is massively involved by inflammatory infiltrate that significantly impairs fetal–maternal exchange and adversely affects fetal wellbeing; H&E-stained section at original magnification, 10×. (**B**): Destruction of the placental barrier is seen as early necrosis (loss of nuclear basophilia) of the villous trophoblast; H&E slide; original magnification, 20×. (**C**): The picture shows the predominant histiocytic component of the inflammatory infiltrate in the intervillous space; positive immunoexpression for CD68 antibody; original magnification, 20×. (**D**): The picture shows the CD3+ T-lymphocytes component of the inflammatory infiltrate in the intervillous space; positive immunoexpression for CD3 antibody; original magnification, 20×. (**E**): The picture shows the CD8+ T-lymphocytes component of the inflammatory infiltrate in the intervillous space; positive immunoexpression for CD8 antibody; original magnification, 40×. (**F**): SARS-CoV-2 placentitis: the picture shows strong positive and confluent immunoexpression in villous trophoblast for SARS-CoV-2 antibody. Placental viral infection triggered massive chronic intervillositis presented in (**A**–**E**); original magnification 40×.

Due to potential risk of infection of medical staff, special precautions were taken, and a special safety procedure was incorporated for these cases. Prior to the gross examination and dissection, whole placentas were fixed in 10% buffered formalin for more than 72 h. Due to the fixation in formalin, taking placental swabs for RT-PCR SARS-CoV-2 was not applicable. The placental examination was performed in accordance with the Amsterdam Placental Workshop Group Consensus Statement for sampling of placental lesions by a perinatal pathologist [23]. For placentas without grossly visible abnormal areas identified and due to positive SARS-CoV-2 maternal status, a submitting protocol was extended for extraplacental membranes and placental parenchyma. When abnormal lesions were identified by a gross examination, further laboratory processes were carried out routinely. Sampled placental sections were fixed in formalin, embedded into paraffin blocks, and stained using hematoxylin and eosin. Immunohistochemistry was automatically processed at the DAKO Autostainer (DAKO Colorado, Inc., Fort Collins, CO, USA) according to the manufacturer’s protocol. A negative control was processed in each run. SARS-CoV-2 immunohistochemistry was performed using a monoclonal antibody against viral nucleocapsid (Invitrogen, ThermoFisher Scientific, Waltham, MA, USA; B46F; 1:200) for each placenta from SARS-CoV-2-positive mothers, including in cases without morphological findings that had been previously reported as associated with COVID-19 maternal infection. When massive chronic intervillositis was suspected on H&E sections, CD3, CD8, and CD68 (X) antibodies were used to confirm the diagnosis. When chronic chorionitis of fetal membranes was suspected (villitis of unknown etiology or chronic chorioamnionitis were not identified in any case of our study group), it was confirmed using CD3 and CD8 antibodies. The microimages in Figure 2 were acquired using a CX43 Olympus microscope with an EPview^TM^ digital camera, version V3.7.2._20200824 (Olympus Soft Imaging Solutions GmbH, Münster, Germany) and using a tablet running EPview application in 300 dpi resolution.

### 2.3. Statistical Analysis

Descriptive data were presented as numbers and percentages for categorical variables, and as the mean, standard deviation, median, and interquartile range (IQ) for numerical variables. The Chi-square or Fisher exact tests were used for the comparison of qualitative variables. The Mann–Whitney U test was used for subgroup analysis of non-normally distributed variables. The data were analyzed using GraphPad Prism 8.0.1. Missing data were excluded from all analyses. The results were considered statistically significant at *p* values of 0.05 or less.

### 2.4. Ethical Approval

The study protocol and publication of fully anonymized data were approved by the Institutional Review Board and Ethics Committee of the Wroclaw Medical University, Poland, approval number KB-444/2021. The data were collected retrospectively; thus, informed consent to participate in the study was not required.

## 3. Results

One-hundred and sixty-one patients were admitted to the Department of Obstetrics and Gynecology and/or the Intensive Care Unit of the University Hospital in Wroclaw because of COVID-19 symptoms and tested positive for SARS-CoV-2. The testing was based on the protocol published by the World Health Organization (WHO). Nasopharyngeal swab specimens were taken from all patients, and SARS-CoV-2 RNA was detected in the samples by reverse-transcription polymerase chain reaction (RT-PCR). One-hundred and thirty-five patients were discharged from hospital after recovery, with ongoing pregnancy. Twenty-six women delivered during the hospital stay. Patients who delivered during hospital stay were aged from 22 to 44 (mean 31). The characteristics of the study population and the infection status of the mother and the neonate are shown in Table 1. A summary of placental pathology findings are presented in Table 2. All pregnancies were single.

**Figure 3:**
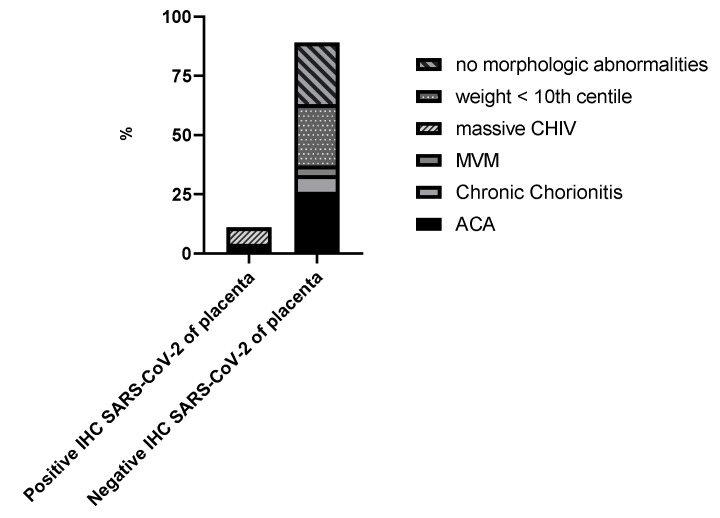
Placental pathology findings in the study. Two patients SARS-CoV-2-positive by IHC of placenta group and 24 patients SARS-CoV-2-negative by IHC of the placenta subgroup. Abbreviations: massive CHIV—massive chronic histiocytic intervillositis; MVM—maternal vascular malperfusion; ACA—acute chorioamnionitis.

Two patients out of twenty-six (7.7%) had the placental infection proven by IHC for SARS-CoV-2. Placentas of 24 patients out of 26 (92.3%) were negative after placental IHC for SARS-CoV-2 examination. Women with SARS-CoV-2-positive placentas had significantly different placental histopathological findings and laboratory results when compared to patients with SARS-CoV-2-negative placentas. Figure 3 presents placental pathology findings for these groups.

Eight placentas out of twenty-six (30.8%) had acute chorioamnionitis (ACA) of fetal membranes. In all cases with ACA, grade 1 of the maternal inflammatory response was diagnosed, whereas the stage varied between 1 and 2. Additionally, all ACA cases were accompanied by acute subchorionitis of the chorionic plate. Two placentas out of twenty-six (7.69%) had mild chronic chorionitis of fetal membranes with CD3/CD8 inflammatory infiltrate highlighted by the immunohistochemistry without morphologic evidence of villitis of unknown etiology, and in one case (3.8%) segmental maternal vascular malperfusion (MVM) manifested by single villous infarcts involving less than 5% of placental parenchymal volume was detected. Small placenta, defined as weight <10th percentile for gestational age, was detected in seven cases (26.9%), though there was no such placenta in the immunohistochemistry SARS-CoV-2-positive subgroup. Six placentas with no histological findings detected in histopathological examination were classified as having no morphological abnormalities (23.1%).

All placentas with positive SARS-CoV-2 status in immunohistochemistry (*n* = 2/2; 100%) had massive chronic histiocytic intervillositis, which represented 7.69% of all placental subgroup in our study. In all these cases, adverse perinatal outcomes were found, one with intrauterine fetal demise and one with low Apgar score. One of the SARS-CoV-2-positive placentas had ACA co-existing. In 6 of 26 cases, other placental abnormalities were also found and included abnormal placental insertion sites or hypercoiling of the umbilical cord in the vast majority of cases. Subsequent low-grade global fetal vascular malperfusion morphologically manifested as small foci of karyorrhectic villi. In any placenta with positive SARS-CoV-2 immunohistochemical status, the fetal vascular malperfusion was not diagnosed.

Only one infant born to a woman with an IHC-positive placenta had the positive result confirmed by RT-PCR in perinatal autopsy via nasopharyngeal swab. In turn, none of the infants born to women with IHC negative placenta had a positive RT-PCR result for a nasopharyngeal swab.

**Figure 4:**
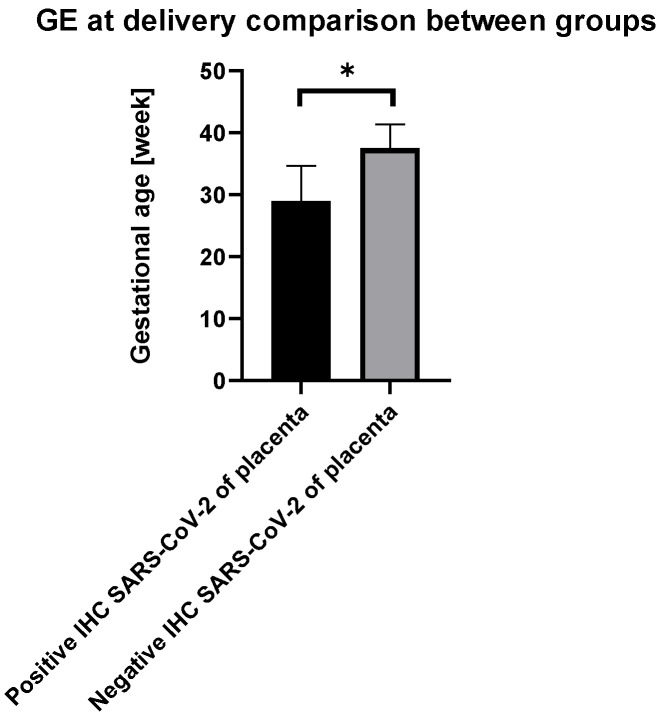
Comparison of week of gestation at delivery between subgroups (*p* = 0.023). Significance code: * < 0.05.

Comparison of gestational week at delivery of women with results of placental IHC for SARS-CoV-2 are presented below (Figure 4).

Of two women with positive placental IHC results for SARS-CoV-2, both gave birth prematurely, in week 25 and week 33 of gestation. In the group of women with negative placental IHC results for SARS-CoV-2, four women gave birth prematurely, at weeks 26, 29, 32, and 34 of gestation (*p* = 0.023).

Nine newborns out of twenty-six had anti-SARS-CoV-2 IgG antibodies measured. A stillborn fetus of a mother with positive placental IHC for SARS-CoV-2 and one infant born to a mother with negative placental IHC for SARS-CoV-2 had anti-SARS-CoV-2 IgG detected. Anti-SARS-CoV-2 IgG was not detected for 6 infants out of 26. For 18 infants, anti-SARS-CoV-2 IgG analysis was not performed.

Results of laboratory tests among subgroups of mothers have been compared. Mothers with positive results of placental IHC for SARS-CoV-2 had higher levels of CRP when compared to the mothers with negative results of placental IHC for SARS-CoV-2, but no statistical significance was found (*p* = 0.726). The comparison of aPTT levels among the mothers also was performed. Mothers with positive placental IHC for SARS-CoV-2 had comparable levels of aPTT with those of mothers with negative results (*p* = 0.667). Mothers with positive of placental IHC for SARS-CoV-2 had lower levels of PLT when compared to the mothers with negative placental IHC for SARS-CoV-2 (*p* = 0.012).

## 4. Discussion

In this retrospective study, we analyzed clinical characteristics of RT-PCR SARS-CoV-2-positive pregnant women and their infants, along with the placental pathology correlation results, including villous trophoblast immunoexpression status for SARS-CoV-2 antibody. These women were admitted to one of the Departments of Obstetrics and Gynecology and/or Intensive Care Units in city of Wroclaw in Poland during the SARS-CoV-2 pandemic in 2021. To obtain more objective results, our investigation correlated histomorphologic findings with proven SARS-CoV-2 infection in the placental tissues. The results have been compared with published analyses [16,20,21,22,24].

The placenta plays a significant role in maintaining immune tolerance to the fetal cells and creates an immunological barrier protecting against pathogens’ entrance. An important contribution of the innate immune system has been proposed recently in the literature: protecting both fetuses and neonates against SARS-CoV-2 infection [25]. There has been reported quite a lot of evidence of SARS-CoV-2 maternal infection during pregnancy [26,27]; nonetheless, reports of probable or proven viral vertical transmission are quite rare [7,20,26,28,29]. Our results were highly consistent with these data, as only 7.7% (*n* = 2/26) of placentas of SARS-CoV-2-positive mothers had viral infection proven by immunohistochemistry in this analysis. Noteworthily, one infant (*n* =1/2; 50%) of a SARS-CoV-2-positive mother with an IHC-positive placenta also had a positive result in RT-PCR (a case confirmed postmortem during perinatal autopsy, as this was the case of intrauterine fetal demise). In parallel, none of the infants born to women with IHC-negative placentas had positive results through RT-PCR of nasopharyngeal swab. Pathological abnormalities most likely not associated with SARS-CoV-2 infection (abnormal umbilical cord insertion or hypercoiling of umbilical cord) were detected in almost half of the placentas, despite maternal SARS-CoV-2 positivity (*n* = 13/26; 50%). However, the SARS-CoV-2 antibody immunoexpression was negative in these cases in our study. Thus, our findings may confirm the strength of the placental barrier, but also, they may suggest a high risk of adverse fetal/neonatal outcomes when it has been damaged by a SARS-CoV-2 infection.

Blumberg et al. reported a situation where vertical transmission from infected mother to infant is possible, when the infection starts 14 days before the delivery. Nevertheless, the infection rate among the infants was very low, as it was in our study. Only one infant had possible SARS-CoV-2 after in utero transmission according to the WHO categorization (positive fetal nasopharyngeal swab by RT-PCR and positive placental tissue by IHC)—detected postmortem, as the infant met intrauterine fetal demise—whereas 25 infants had negative RT-PCR results [7].

Our results demonstrated significantly different pathology results in placentas from IHC-proven SARS-CoV-2-infected mothers compared to the subgroup without evidence of infection via placental IHC. Moreover, in all cases with SARS-CoV-2 placental infection proven by immunohistochemistry, a severe placental pathology was diagnosed, consistent with massive chronic histiocytic intervillositis. This placental entity has been reported also by others as having a possible association with maternal SARS-CoV-2 infection and may represent SARS-CoV-2 placentitis [20,26]. Massive CHIV is a rare chronic inflammatory lesion of the placenta with the involvement of >50% of the intervillous space by mononuclear cells; it is likely associated with autoimmune maternal disease. As inflammatory lesions predominantly are localized in the maternal–fetal interface, the presence of massive CHIV significantly impairs the fetal–placental exchange, often leading to poor perinatal outcomes, including intrauterine fetal death. This entity is strongly associated with recurrent abortion, early spontaneous abortion, fetal growth restriction, and fetal loss. Massive CHIV can be morphologically accompanied by an increase in perivillous fibrin deposition, which is associated with adverse pregnancy outcomes [11,30]. Evidence from the era before the SARS-CoV-2 pandemic showed very high rates of recurrence for CHIV at the level of 67–100% depending on the source [31], which makes this placental entity highly dangerous not only in present, but for future pregnancies as well. Massive CHIV was detected in our study in 100% of placentas (*n* = 2/2; 2/26) with placental IHC SARS-CoV-2 positivity, and was etiologically responsible for both adverse perinatal outcomes observed in our study: the intrauterine fetal demise and low Apgar score of the neonate. Thus, based on our results, we support the suggestion reported in other studies that massive CHIV can represent one of the morphological placental manifestations of SARS-CoV-2 placentitis and may be a marker of potential vertical transmission [20]. Further investigations need to be conducted with careful clinical–pathological correlation by specialized perinatal interdisciplinary teams to assess the significance and potential associations of less specific placental pathologies with maternal SARS-CoV-2 infection.

The pathology findings differ in several aspects compared to other studies. We did not observe any case of villitis of unknown etiology (VUE) in the entire placenta group in our analysis, regardless of IHC SARS-CoV-2 placental status. To date, VUE has been reported as being associated with maternal SARS-CoV-2 infection with proven placental infection in 14.7% of cases [12]. Yet, our analysis revealed mild chronic chorionitis of fetal membranes, as proven by CD3/CD8 immunohistochemistry, in 7.69% of placentas from infected mothers. Chronic chorionitis might be a morphological component of VUE [11], but the reasons for the presence of mild chronic chorionitis only without villitis in our analysis remain unclear, and this aspect requires further research. According to a large well-designed review that included studies with the evidence not only of SARS-CoV-2 maternal infection but also with infection of the placental tissues, the most commonly reported feature (37.8%) among histopathological findings is maternal vascular malperfusion [12]. In our results, segmental maternal vascular malperfusion (MVM) was detected in one case (3.8%; 1/26), and it was manifested by single villous infarcts involving less than 5% of placental parenchymal volume. There are likely several potential reasons for these differences in histopathology findings. The most important ones might be small sizes of the groups studied worldwide, insufficient clinical–pathological correlations, different populations being included, or, simply, there still being little evidence for the perinatal aspects of SARS-CoV-2 infection.

In 26.9% of cases (*n* = 7) of SARS-CoV-2-positive mothers, a small placenta was detected in the pathology examination, despite negative placental IHC immunoexpression. The normal fetal–placental weight ratios, the lack of other accompanying placental morphological findings in vast majority of these cases, and the absence of evidence of small placentas in the IHC SARS-CoV-2-positive subgroup, suggest that the real small for gestational age placentas occurred due to rather than in association with the SARS-CoV-2 etiology and/or placental insufficiency.

One newborn born to a mother with positive placental IHC had the anti-SARS-CoV-2 IgG present, and one newborn of a mother with negative placental IHC for SARS-CoV-2 also had the anti-SARS-CoV-2 IgG. The research on anti-SARS-CoV-2 antibodies conducted by Bwire et al. focused on antibody transmission from mother to infant. In the study, 18.8% of infants born to positive mothers had elevated levels of anti-SARS-CoV-2 IgG/IgM antibodies, which makes the vertical transplacental transmission of antibodies possible, although rare [32]. Another study on infant anti-SARS-CoV-2 antibodies was described by Anna F. Cavaliere et al. [33]. They described a situation in which anti-SARS-CoV-2 IgG antibodies were present in infant blood and IgM antibodies were not. In the study described by Zeng, the RT-PCR results of newborn throat swabs were SARS-CoV-2 negative, IgG antibodies were detected, and IgM antibodies were not found in the infant’s blood [21].

Giannis et al. reported that abnormalities in a blood coagulation in SARS-CoV-2-positive mothers were found. All of the patients were receiving low-molecular-weight heparin [34]. The statistical significance was found when PLT 10^3^/uL between subgroups was compared. Mothers with positive placental IHC had lower platelet counts than patients with negative placental IHC. The significantly lower platelet count noted by G.Lippi et al. was also identified in patients with severe disease [22]. Although no statistical significance was found when other laboratory results were compared, these two subgroups of women had slightly different outcomes.

The small sample size presented in our manuscript corresponds to other published studies on clinico-pathological correlations in SARS-CoV-2-positive pregnant women, where the number of analyzed cases varied from 1 to 33 [16,20,21,22,35]. According with the largest reported review comprising placental morphology lesions, to date, there were only 56 studies reporting histopathological findings on second and third trimester placentas, for a total of 1008 placental cases [12]. This gives 18 assessed placentas per study. Thus, the sample size in our study (*n* = 26 cases) was within the worldwide average. When including studies with SARS-CoV-2 infection in placental tissues proven by currently accepted standards and a proper morphological assessment, a total of only 36 placentas were globally reported on with the use of these requirements. Perinatal aspects of SARS-CoV-2 infection of pregnancy with correlating placental histopathological examination performed by a qualified perinatal pathologist were reported mostly in the case series. These data show that completing the reliable evidence about the association between maternal SARS-CoV-2 infection and placental function reflected in placental morphology is difficult and needs interdisciplinary efforts. We believe that this analysis will help to decrease the knowledge gap on the impacts of COVID-19 in pregnant women, and of maternal SARS-CoV-2 infection on the placenta and fetal/neonatal outcomes. Only growing evidence might help in facing the perinatal cases with this globally novel viral infection.

## 5. Conclusions

In conclusion, this study on maternal-neonatal outcomes and placental pathology correlations with SARS-CoV-2 immunoprofile analysis in placental tissues showed a strong association between proven placental infection and severe inflammatory placental lesions consistent with massive chronic histiocytic intervillositis in SARS-CoV-2-positive mothers, and low rates of vertical transmission. The presence of anti-SARS-CoV-2 IgG in sera of the newborns of infected mothers was not associated with the positive placental immunoexpression of SARS-CoV-2 antibodies. Further studies are warranted to confirm these findings in larger cohorts and/or prospective settings. They should be conducted in different populations with a comparison group of pregnant women without proven SARS-CoV-2 infection. Clinical data and placental pathology correlation might be substantial in the process of understanding the course of the infection, implications for intrauterine fetal condition, and potential risk for vertical transmission in SARS-CoV-2-positive women.

## Figures and Tables

**Table 1:** Characteristics of the 26 patients enrolled in the study.

Variables	
Weeks of gestation at delivery	37 (Mean), 25 week (Minimum), 41 (Maximum),
Delivery route	*n* = 6, 23% Vaginal; *n* = 19, 73% Cesarean; *n* = 1, 4% Spontaneous Abortion
PCR of amniotic fluid	*n* = 1, 4% Positive; *n* = 11, 42% Negative; *n* = 14, 54% Not Confirmed
PCR of infant	*n* = 1, 4% Positive; *n* = 25, 96% Negative
IHC of placenta	*n* = 2, 8% Positive; *n* = 24, 92% Negative
Maternal Anti-SARS-CoV-2 IgG	*n* = 3, 11% Positive; *n* = 5, 19% Negative; *n* = 18, 70% Not examined
Infant Anti-SARS-CoV-2 IgG	*n* = 3; 12% Positive; *n* = 6, 23% Negative; *n* = 17, 65% Not examined

**Table 2:** Summary of placental pathology findings of 26 patients enrolled in the study.

Placental Diagnosis	Further Morphological Findings	Number of Cases Detected in Placental Subgroup; No (%)
Massive Chronic Histiocytic Intervillositis (MCHI)	All cases of MCHI in the study were detected in IHC SARS-CoV-2-positive placenta subgroup. Massive inflammatory infiltrate involving >30% percent of placental parenchyma was highlighted by CD68/CD3/CD8/CD138 panel of antibodies.	2/26 (7.69%)
Acute chorioamnionitis (ACA) of fetal membranes	Grade 1 and stage 1-2 of maternal inflammatory response was detected in all of ACA cases	8/26 (30.77%)
Placenta weight < 10th centile	All cases of small placenta were detected in IHC SARS-CoV-2-negative placenta subgroup	7/26 (26.92%)
Fetal vascular malperfusion (FVM)	Detected FVM included abnormal placental insertion site or hypercoiling of the umbilical cord with subsequent low-grade global FVM morphologically manifested as small foci of karyorrhectic villi	6/26 (23.07%)
Chronic chorionitis of fetal membranes	Mild chronic chorionitis of fetal membranes with CD3/CD8-positive inflammatory infiltrate was diagnosed, without morphologic evidence of villitis of unknown etiology	2/26 (7.69%)
Maternal vascular malperfusion (MVM)	One case of segmental MVM manifested by single villous infarct involving <5% of placental parenchyma was found	1/26 (3.84%)
No morphological abnormalities	All cases with no morphologic abnormalities were detected in IHC-SARS-CoV-2-negative placental subgroup	7/26 (26.92%)
Additional notes regarding positive placental SARS-CoV-2 status in IHC	All placentas with positive SARS-CoV-2 status in IHC (n = 2/2; 100%) had MCHI. In all these cases adverse perinatal outcomes were found, one with intrauterine fetal demise and one with low Apgar score. One of SARS-CoV-2-positive placentas had ACA co-existing. Morphologic features of MVM or FVM were not detected in that subgroup. In any placenta with negative IHC SARS-CoV-2 status, morphologic features of chronic histiocytic intervillositis were not diagnosed.	

Abbreviations: MCHI, massive chronic histiocytic intervillositis; IHC, immunohistochemistry; ACA, acute chorioamnionitis; FVM, fetal vascular malperfusion; MVM, maternal vascular malperfusion.

## Data Availability

The datasets used and/or analyzed during the current study are available from the corresponding author upon reasonable request.
